# Artificial intelligence predicts clinically relevant atrial high-rate episodes in patients with cardiac implantable electronic devices

**DOI:** 10.1038/s41598-021-03914-4

**Published:** 2022-01-07

**Authors:** Min Kim, Younghyun Kang, Seng Chan You, Hyung-Deuk Park, Sang-Soo Lee, Tae-Hoon Kim, Hee Tae Yu, Eue-Keun Choi, Hyoung-Seob Park, Junbeom Park, Young Soo Lee, Ki-Woon Kang, Jaemin Shim, Jung-Hoon Sung, Il-Young Oh, Jong Sung Park, Boyoung Joung

**Affiliations:** 1 Division of Cardiology, Chungbuk National University Hospital, Cheongju, Korea; 2 Medtronic Korea, Seoul, Korea; 3 Division of Cardiology, Department of Internal Medicine, Severance Cardiovascular Hospital, Yonsei University College of Medicine, 50-1 Yonsei-ro, Seodaemun-gu, Seoul, 03722 Republic of Korea; 4 Division of Cardiology, Dong-A University Hospital, 26 Daesingongwon-ro, Seo-gu, Busan, 49201 Republic of Korea; 5 Department of Preventive Medicine and Public Health, Yonsei University College of Medicine, Seoul, Korea; 6 Department of Cardiology, Seoul National University Hospital, Seoul, Korea; 7 Division of Cardiology, Keimyung University Hospital, Daegu, Korea; 8 Department of Cardiology, Ewha Womans University Hospital, Seoul, Korea; 9 Division of Cardiology, Daegu Catholic University Hospital, Daegu, Korea; 10 Division of Cardiology, Eulji University Hospital, Daejeon, Korea; 11 Department of Cardiology, Korea University Hospital, Seoul, Korea; 12 Department of Cardiology, CHA Bundang University Hospital, Seongnam, Korea; 13 Department of Cardiology, Seoul National University Bundang Hospital, Seongnam, Korea

**Keywords:** Cardiology, Risk factors

## Abstract

To assess the utility of machine learning (ML) algorithms in predicting clinically relevant atrial high-rate episodes (AHREs), which can be recorded by a pacemaker. We aimed to develop ML-based models to predict clinically relevant AHREs based on the clinical parameters of patients with implanted pacemakers in comparison to logistic regression (LR). We included 721 patients without known atrial fibrillation or atrial flutter from a prospective multicenter (11 tertiary hospitals) registry comprising all geographical regions of Korea from September 2017 to July 2020. Predictive models of clinically relevant AHREs were developed using the random forest (RF) algorithm, support vector machine (SVM) algorithm, and extreme gradient boosting (XGB) algorithm. Model prediction training was conducted by seven hospitals, and model performance was evaluated using data from four hospitals. During a median follow-up of 18 months, clinically relevant AHREs were noted in 104 patients (14.4%). The three ML-based models improved the discrimination of the AHREs (area under the receiver operating characteristic curve: RF: 0.742, SVM: 0.675, and XGB: 0.745 vs. LR: 0.669). The XGB model had a greater resolution in the Brier score (RF: 0.008, SVM: 0.008, and XGB: 0.021 vs. LR: 0.013) than the other models. The use of the ML-based models in patient classification was associated with improved prediction of clinically relevant AHREs after pacemaker implantation.

## Introduction

Multiple clinical trials have demonstrated that longer atrial high-rate episodes (AHREs) are associated with an increased risk of atrial fibrillation (AF), ischemic stroke, and adverse cardiovascular outcomes^1–5^. Therefore, the current European Society of Cardiology (ESC) guidelines recommend comprehensive cardiovascular evaluation, including the stroke risk, medical comorbidities, and risk factors, in patients with AHREs detected by implanted devices. Notably, in patients with a longer duration of AHREs, more intensive monitoring can be more useful^6,7^. Previous clinical studies have demonstrated multiple predictors of AHREs in patients with pacemakers^1,4,8–10^. However, these inconsistent predictors paradoxically indicate that the etiology is ambiguous. Therefore, precisely estimating clinically relevant AHREs is an important part of optimal patient management after pacemaker implantation. Machine learning (ML) offers a computational and alternative approach to standard predictive modeling that recognizes complex characteristics within data^11^. To date, there have been several investigations of ML algorithms for coronary artery disease genetics^12^, cardiac resynchronization therapy outcomes, such as mortality and heart failure (HF)-related hospitalization^13^, HF with preserved ejection fraction^14^, and estimation of ventricular tachycardia recurrence and mortality after catheter ablation^15^ in the cardiology era. We hypothesized that ML algorithms can produce a predictive model for clinically relevant AHREs in individual patients that can be more useful than previously reported predictors. Herein, we sought to determine which class of ML algorithms has the highest predictive accuracy using data from a prospective multicenter registry.

## Materials and methods

### Study design

The cohort of patients in this study was derived from the evaluation of the **A**trial **F**ibrillation occurrence in patients after **P**acemaker implantation (**AF**-**P**acemaker study), a prospective, multicenter, observational registry study performed in patients with AF aged > 18 years attending any of the 11 tertiary hospital centers comprising all geographical regions of Republic of Korea. The study enrollment period started in September 2017 and ended in July 2020.

The **AF**-**P**acemaker study aimed to investigate the occurrence and management (including ablation therapy) of device-detected AF episodes in patients with pacemaker implants through a prospective, non-randomized, non-blinded, observational, multicenter design. The study was conducted in compliance with the ethical rules of the Declaration of Helsinki as a statement of ethical principles for medical research involving human subjects by the World Medical Association and approved by the Institutional Review Board of Yonsei University Health System (1-2017-0008). This study was registered at ClinicalTrials.gov (NCT03303872, First posted on October 6, 2017). The ethics committees of all 11 tertiary hospitals (Severance Hospital, Seoul National University Bundang Hospital, Seoul National University Hospital, Donga University Medical Center, Keimyung University Hospital, Ewha Womans University Medical Center, Daegu Catholic University Medical Center, Korea University Medical Center, Eulji University Hospital, CHA Bundang Medical Center, and Kangneung Asan Medical Center) approved this study, and all patients provided informed consent for their inclusion.

### Study population

The study population included patients (1) eligible for permanent pacemaker implantation according to the guidelines on cardiac pacemaker implantation for sick sinus syndrome (sinus bradycardia, sinus pause of ≥ 3 s, tachy-brady syndrome, sinus node dysfunction, and chronotropic incompetence) or atrioventricular block (high-degree/complete atrioventricular block), (2) with an atrial sensing capability.

**Figure 1:**
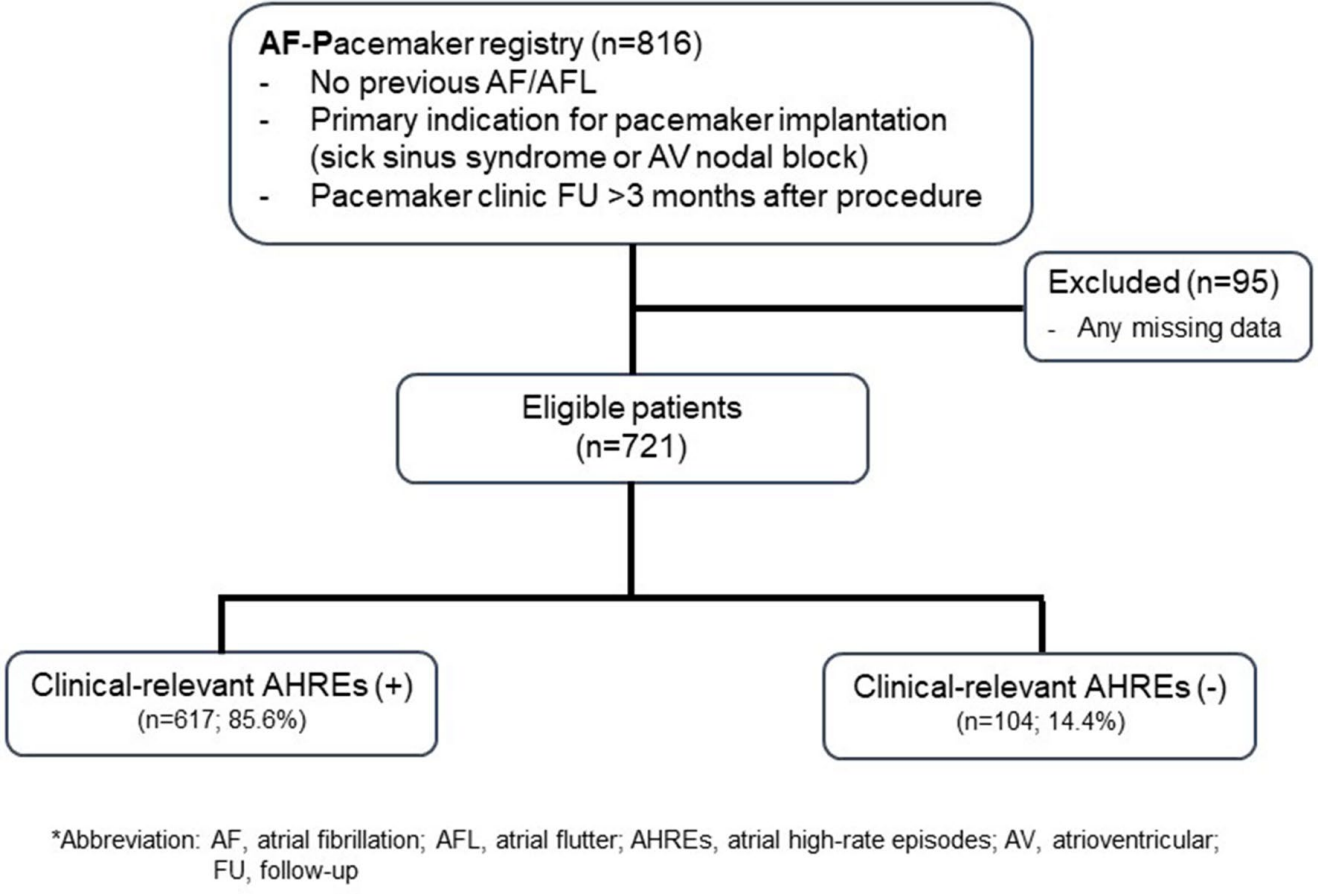
Study population selection process.

A total of 816 consecutive patients who were implanted with a St. Jude Medical dual-chamber rate-adaptive pacemaker (Assurity PM2240) with stored electrogram capabilities were enrolled. The pacemakers were incorporated with bipolar atrial and ventricular leads (Tendril MRI LPA1200M, Isoflex Optim 1944/1948, and Tendril ST Optim 1888TC) in all patients. The atrial and ventricular leads were placed in the right atrial appendage and right ventricular apex, respectively. We excluded patients with severe liver dysfunction (aspartate aminotransaminase/alanine aminotransferase level ≥ 3 times the normal upper limit) or severe renal dysfunction (serum creatinine level of ≥ 3.5 mg/dL or creatinine clearance of ≤ 30 mL/min), including conditions requiring dialysis; pregnant or lactating patients; or those malignant cancer, dilated cardiomyopathy, hypertrophic cardiomyopathy, severe valvular heart disease, or life expectancy of ≤ 12 months from enrollment. Further, 95 patients with missing data for analysis were excluded (Fig. 1). Based on the available data from 721 patients567 patients were used for model development and 154 for validation.

### Data collection, Pacemaker programming and AHREs detection

For the **AF**-**P**acemaker study, data were collected by independent clinical research coordinators via Web-based case report forms on the Internet-based Clinical Research and Trial management system (iCReaT), a data management system established by the Centers for Disease Control and Prevention, Ministry of Health and Welfare, Republic of Korea (iCReaT Study No. C170004). Each center could see its data and those of the other participating centers.

An AHRE detection rate of 220 beats/min was programmed, and storage of up to four atrial electrograms of 12-s duration on automatic detection of AHREs was activated. At every visit, the longest duration of all AHREs was ascertained. The longest AHRE (> 6 min) was defined as clinically relevant AHREs. Device interrogation information was obtained at regular clinic visits every 6 months after pacemaker implantation. An interval of up to 3 months before and after each clinic visit was allowed. The AHREs were interrogated to compare electrocardiogram (ECG) traces and pacemaker AHREs data at the end of the ambulatory monitoring period. The clinicians were blinded to the atrial diagnostic data. A detailed study protocol has been published previously^16^.

### Model development

#### Predictive feature selection

We collected 28 variables available before the time of pacemaker implantation, including baseline patient demographic characteristics, clinical information, medications, and 12-lead ECG, laboratory examination, Holter monitoring, treadmill test, and transthoracic echocardiography findings. The predictors were age, sex, body mass index, current or former smoking status, current or former alcohol consumption, baseline heart rate, baseline systolic blood pressure, baseline diastolic blood pressure, indications for pacemaker implantation (sinus node dysfunction or atrioventricular node disease), estimated glomerular filtration rate (eGFR), left atrium (LA) diameter, left ventricular ejection fraction (LVEF), QRS duration, corrected QT (QTc) interval, presence of HF, hypertension, diabetes mellitus, prior stroke or transient ischemic attack (TIA), vascular disease, chronic kidney disease, presence of ventricular premature contraction, presence of atrial premature contraction, dyslipidemia, and medications, including renin‒angiotensin‒aldosterone system blockers, beta adrenergic receptor blockers, calcium channel blockers, diuretics, and statins. Continuous variables were evaluated using point-biserial correlation coefficients^17^ in relation to the clinically relevant AHREs, and the correlation of discrete variables was measured using Cramér’s V^18^. Considering the two indicators, variables showing a low degree of correlation value using the knee point with the clinically relevant AHREs were excluded from the modeling process (Supplementary Table 1 and Supplementary Fig. 1). In addition, all continuous variables were normalized using Z-score normalization. Finally, the predictive models were constructed with subset data consisting of 10 variables based on model performance index for maximum prediction performance, excluding 18 variables, as shown in Supplementary Table 2.

#### Imbalanced data preprocessing

When a predictive model is trained on imbalanced data, it tends to classify patterns biased into majority classes and ignores the characteristics of minority classes. Considering the imbalance problem of the study population, we applied a balancing method using the synthetic minority oversampling technique (SMOTE)^19^, and the minority class (clinically relevant AHREs) was oversampled. Clinically relevant AHREs were noted in 14.4% of the original dataset but in 30.8% of the SMOTE-balanced dataset after oversampling.

#### Model derivation and algorithms

**Figure 2:**
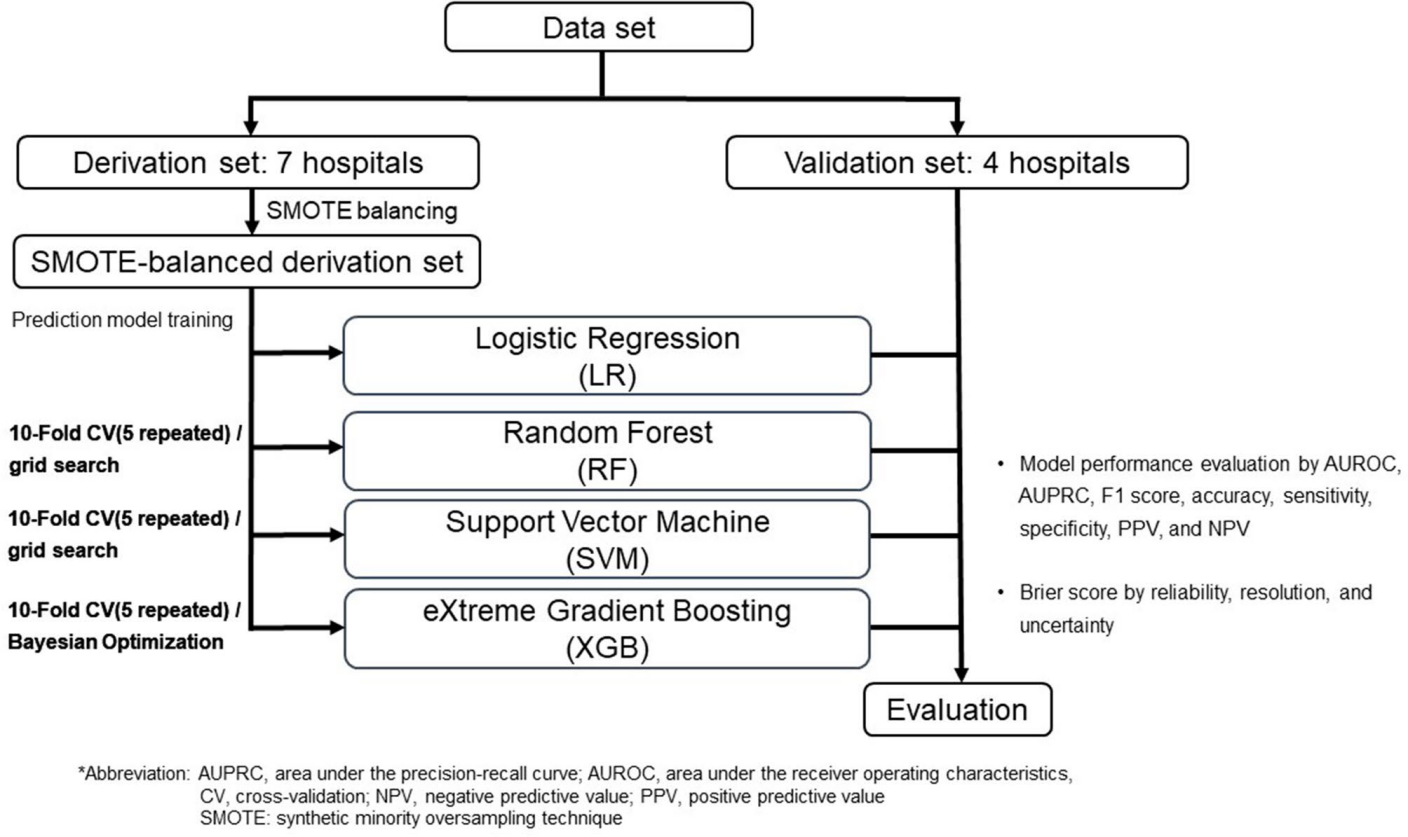
Flow diagram for the modeling process.

To develop ML algorithms, we divided the study population into a training set, in which the prediction algorithm for clinically relevant AHREs was derived, and a test set, in which the algorithm was evaluated based on hospital units. The training set was derived from the patient data of seven hospitals and the test set from the patient data of the four remaining hospitals. We developed three ML-based predictive models: the random forest (RF)^20^ algorithm, support vector machine (SVM)^21^ algorithm, and extreme gradient boosting (XGB)^22^ algorithm, in comparison to conventional logistic regression (Fig. 2).

RF algorithm is an ensemble learning method that creates multiple prediction results by multiple decision trees and determine the outcome by majority vote from decision tree results. We developed our RF model with 300 decision trees (Supplementary Fig. 2) and the average tree depth is 16 ± 2.1 (mean ± SD). SVM is an algorithm that finds a hyperplane that divides two categories of data in a multi-dimensional space of data. Lastly, XGB is an ensemble algorithm to create a strong decision tree classifier by combining weak tree classifiers built sequentially that each subsequent tree is trained from residuals of the previous tree to reduce its error.

We applied five repetitions of the tenfold cross validation (CV)^23^ method to validate predictive models with hyperparameters tuned by a grid search algorithm for RF and SVM, and Bayesian optimization^24^ algorithm for XGB. To determine how each variable affects the prediction of outcome, we calculated the feature importance of individual variables using the average absolute deviation by sensitivity analysis for each model^25^.

### Statistical analysis

Continuous variables were presented as means ± standard deviations for normally distributed values or medians and interquartile intervals for non-normally distributed values and categorical variables as numbers and percentages in each group. The baseline characteristics of the two groups were compared using Student’s t-test or Wilcoxon test for continuous variables and Pearson’s χ^2^ test or Fisher’s exact test for categorical variables. To evaluate the model discrimination and accuracy, we measured the area under the receiver operating characteristic (AUROC) curve, area under the precision–recall curve (AUPRC), F1-score, accuracy, sensitivity, specificity, negative predictive value (NPV), and positive predictive value (PPV) and calculated the Brier score^26^, including reliability, resolution, and uncertainty. All analyses were performed using R (version 4.0.2; R Foundation for Statistical Computing, Vienna, Austria). The dedicated R packets and versions to validate and apply the 3 ML models has been published in GitHub (RF, R package version 4.6–14 https://CRAN.R-project.org/package=randomForest; SVM, R package version 0.9–29 https://CRAN.R-project.org/package=kernlab; XGB, R package version 1.4.1.1. https://CRAN.R-project.org/package=xgboost). Statistical significance was set at* p* values of < 0.05.

## Results

Between September 2017 and July 2020, 721 patients were eligible for inclusion; their median age was 73 years, and 61.5% were women. The median follow-up duration was 18 months. Atrioventricular node disease (56.7%) was the most common indication for cardiac implantable electronic device implantation, followed by sinus node dysfunction (43.3%). A total of 104 patients (14.4%) experienced AHREs lasting for > 6 min, which were defined as clinically relevant AHREs in this study. The patients who experienced clinically relevant AHREs were significantly more likely to have a higher rate of sinus node dysfunction for pacemaker implantation, experience > 1% atrial premature contraction on pre-procedural Holter monitoring, use beta-adrenergic receptor blockers, and have a shorter QRS duration than those who did not experience clinically relevant AHREs (Table 1). The derivation set consisted of 567 patients from seven hospitals, and the validation set consisted of 154 patients from four hospitals. Supplementary Table 3 shows the characteristics of these two sets. The prevalence of former or current smokers and alcohol consumers and dyslipidemia, baseline systolic blood pressure, and LA diameter on echocardiography were higher in the derivation set. Further, this set of patients had lower baseline eGFR and LVEF and shorter QRS duration and QTc interval on electrocardiography.

**Table 1:** Baseline characteristics of the patients with and without clinically relevant AHREs.

Variables	Total (n = 721)	Clinically relevant AHREs (-) (n = 617)	Clinically relevant AHREs ( +) (n = 104)	*p* value
**Demographic**				
Age, (years)	73 (65, 79)	73 (65, 78)	74 (68, 80)	0.155
Male, n (%)	285 (39.5)	242 (39.2)	43 (41.3)	0.763
Body mass index, (kg/m^2^)	24.1 (22.2, 26.2)	24.2 (22.2, 26.2)	23.9 (22.1, 26.1)	0.566
Smoking, n (%)				
Former/Current	90 (12.5)	78 (12.6)	12 (11.5)	0.877
Alcohol, n (%)	93 (12.9)	80 (13.0)	13 (12.5)	1.000
Former/Current				
**Clinical**				
Heart failure, n (%)	26 (3.6)	23 (3.7)	3 (2.9)	1.000
Hypertension, n (%)	484 (67.1)	416 (67.4)	68 (65.4)	0.767
Diabetes, n (%)	196 (27.2)	173 (28.0)	23 (22.1)	0.256
Prior stroke/TIA, n (%)	80 (11.1)	64 (10.4)	16 (15.4)	0.181
Vascular disease, n (%)	71 (9.8)	58 (9.4)	13 (12.5)	0.422
Dyslipidemia, n (%)	222 (30.8)	197 (31.9)	25 (24.0)	0.134
Chronic kidney disease, n (%)	59 (8.2)	50 (8.1)	9 (8.7)	1.000
**CHA**_**2**_**DS**_**2**_VAS score*	3 (2, 4)	3 (2, 4)	3 (2, 4)	0.454
**CHA**_**2**_**DS**_**2**_**VAS score, group**				0.955
0, n (%)	21 (2.9)	18 (2.9)	3 (2.9)	
1, n (%)	104 (14.4)	90 (14.6)	14 (13.5)	
≥ 2, n (%)	596 (82.7)	509 (82.5)	87 (83.7)	
Pacemaker indication				< 0.001
Sick sinus syndrome, n (%)	312 (43.3)	248 (40.2)	64 (61.5)	
AV block, n (%)	409 (56.7)	369 (59.8)	40 (38.5)	
Baseline systolic blood pressure, (mmHg)	135 (121, 148)	135 (122, 148)	133 (120, 145)	0.174
Baseline diastolic blood pressure, (mmHg)	71 (64, 80)	71 (64, 80)	72 (63, 80)	0.735
Baseline heart rate, (/min)	60 (50, 72)	60 (50, 72)	60 (50, 72)	0.954
Baseline eGFR, (mL/min/1.73 m^2^)	78.0 (62.0, 91.0)	77.0 (63.0, 92.0)	80.5 (62.0, 88.3)	0.894
**Electrocardiogram**				
QRS duration, (ms)	106 (90, 142)	108 (90, 144)	98 (88, 134)	0.038
QTc interval, (ms)	455 (422, 488)	455 (423, 488)	451 (420, 477)	0.301
**Echocardiography**				
LA diameter, (mm)	40 (36, 45)	40 (35, 45)	42 (37, 45)	0.094
LVEF, (%)	65 (60, 70)	65 (60, 70)	65 (60, 70)	0.868
**Holter recording**				
APC > 1% at pre-implantation, n (%)	53 (7.4)	38 (6.2)	15 (14.4)	0.005
VPC > 1% at pre-implantation, n (%)	49 (6.8)	39 (6.3)	10 (9.6)	0.306
**Medications**				
ARB/ACEi, n (%)	312 (43.3)	273 (44.2)	39 (37.5)	0.239
Beta adrenergic receptor blocker, n (%)	112 (15.5)	84 (13.6)	28 (26.9)	0.001
Calcium channel blocker, n (%)	224 (31.1)	194 (31.4)	30 (28.8)	0.678
Statin, n (%)	306 (42.4)	267 (43.3)	39 (37.5)	0.320
Diuretics, n (%)	162 (22.5)	139 (22.5)	23 (22.1)	1.000

The data are presented as number (%), median [IQR].

*ACEi* angiotensin-converting-enzyme inhibitor, *AHREs* atrial high-rate episodes, *APC* atrial premature complex, *ARB* angiotensin receptor blocker, *AV* atrioventricular, *GFR* glomerular filtration rate, *LA* left atrium, *LVEF* left ventricular ejection fraction, *QTc* corrected QT, *TIA* transient ischemic attack, *VPC* ventricular premature complex.

*The CHA_2_DS_2_-VAS score is a measure of the risk of stroke in patients with atrial fibrillation, with scoring ranging from 0 to 9 and higher scores indicating greater risk. Congestive heart failure, hypertension, age 75 years or older (doubled), diabetes, stroke (doubled), vascular disease, age 65 to 74 years, sex category (female).

### Model performance

**Figure 3:**
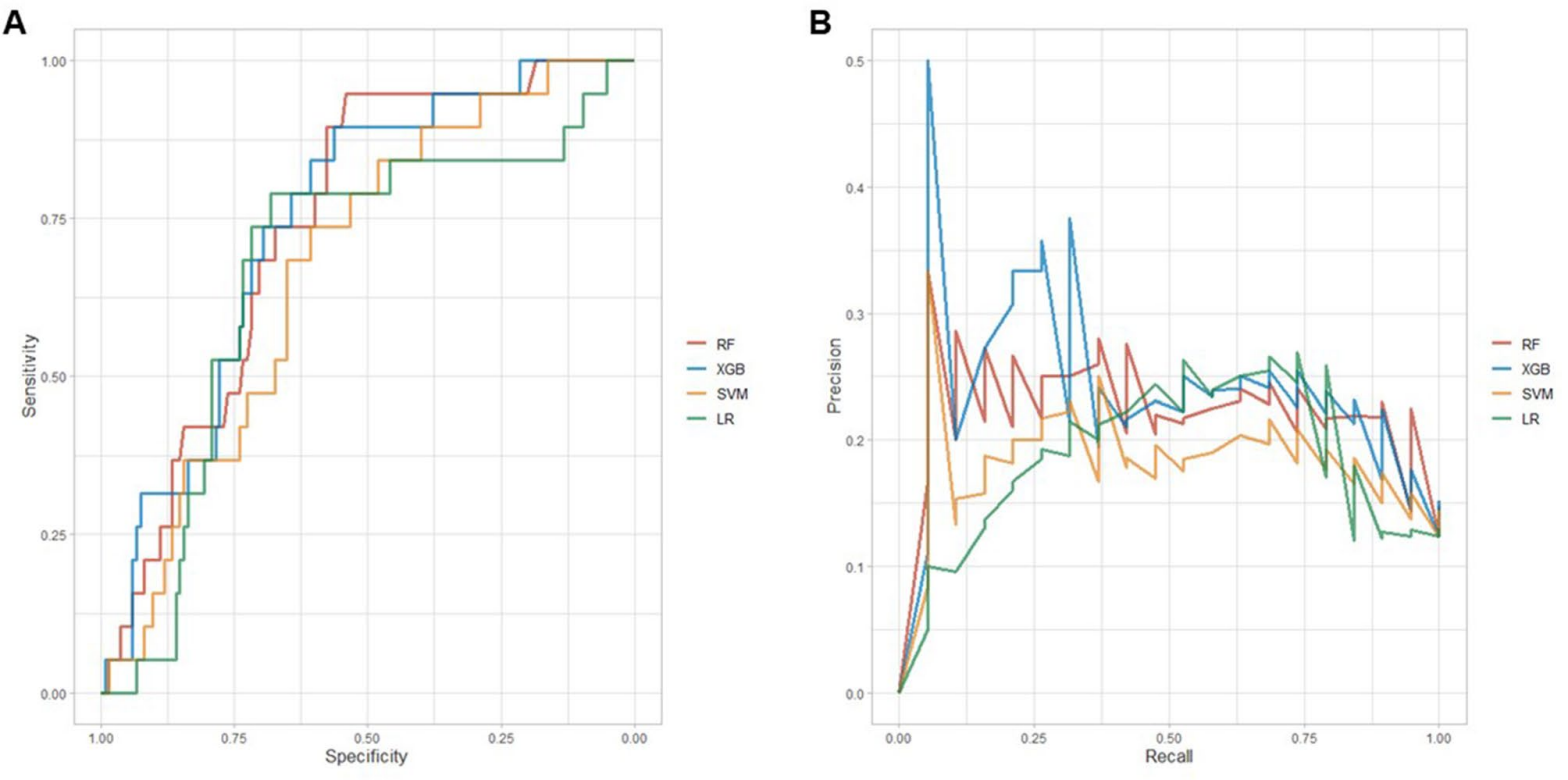
Receiver operating characteristic curve analysis (**A**) and precision–recall curve analysis (**B**) for each model.

In the ML-based models, improvement in discrimination was achieved using the same data of the validation set (Table 2). The AUROCs achieved by each model was 0.742 for the RF algorithm, 0.675 for the SVM algorithm, and 0.745 for the XGB algorithm, which were numerically higher than 0.669 for logistic regression. The AUPRC and F1-score also improved, especially those for the XGB algorithm. The ROC and PRC curve plots are shown in Fig. 3. Using the Brier score, we achieved better performance in all three models based on the reliability, with lower values indicating higher agreement between observed and predicted risks, in comparison with logistic regression. The XGB algorithm had a higher resolution value, which indicated a prediction more accurate than that of the other algorithms across the spectrum risk.

**Table 2:** Performance characteristics of models in the validation set for predicting clinically relevant AHREs in patients with pacemaker.

	Logistic regression	RF	SVM	XGB
**Model performance**				
AUROC (95% CI)	0.669 (0.536–0.803)	0.742 (0.637–0.835)	0.675 (0.561–0.789)	0.745 (0.631–0.847)
AUPRC (95% CI)	0.182 (0.104–0.274)	0.224 (0.119–0.397)	0.182 (0.102–0.337)	0.240 (0.125–0.424)
F1 score (95% CI)	0.853 (0.783–0.881)	0.888 (0.845–0.925)	0.865 (0.821–0.905)	0.896 (0.857–0.931)
Accuracy (95% CI)	0.753 (0.677–0.819)	0.805 (0.734–0.865)	0.773 (0.698–0.836)	0.818 (0.748–0.876)
Sensitivity (95% CI)	0.815 (0.739–0.876)	0.881 (0.815–0.931)	0.830 (0.755–0.889)	0.889 (0.823–0.936)
Specificity (95% CI)	0.316 (0.126–0.566)	0.263 (0.091–0.512)	0.368 (0.163–0.616)	0.316 (0.126–0.566)
PPV (95% CI)	0.194 (0.074–0.375)	0.238 (0.082–0.472)	0.233 (0.099–0.423)	0.286 (0.113–0.522)
NPV (95% CI)	0.894 (0.824–0.943)	0.895 (0.830–0.941)	0.903 (0.837–0.949)	0.902 (0.839–0.947)
**Brier score**				
Overall	0.181	0.138	0.158	0.141
Reliability	0.086	0.038	0.058	0.054
Resolution	0.013	0.008	0.008	0.021
Uncertainty	0.108	0.108	0.108	0.108

*AHREs* atrial high-rate episodes, *AUPRC* area under the precision-recall curve, *AUROC* area under receiver operating characteristic, *CI* confidence interval, *NPV* negative predictive value, *PPV* positive predictive value, *RF* random forest, *SVM* support vector machine, *XGB* extreme gradient boosting.

### Feature importance of the individual variables

**Figure 4:**
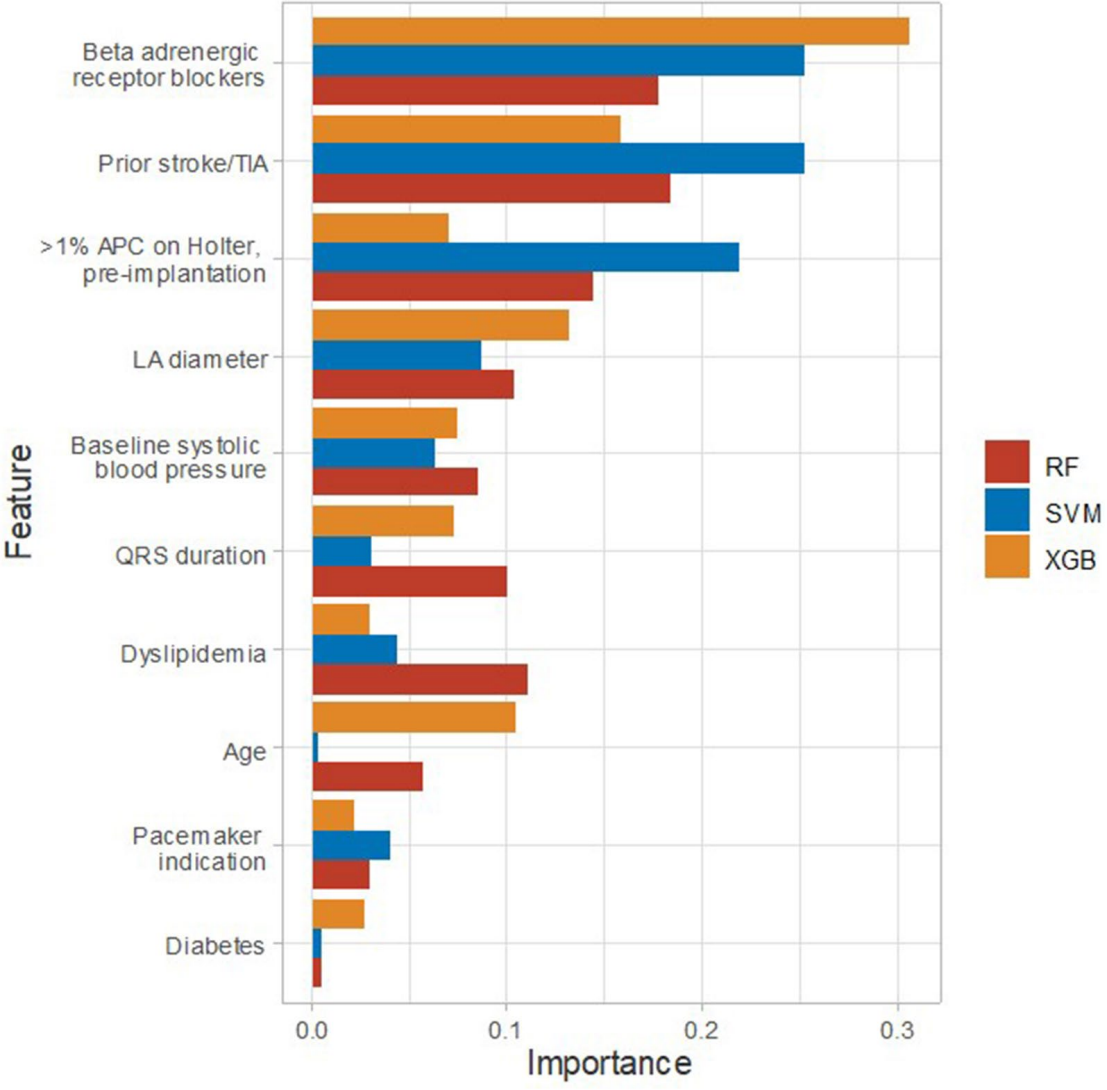
Feature importance index plot for each model.

We observed that beta-adrenergic receptor blocker use, prior stroke or TIA, LA diameter, and > 1% atrial premature contraction on Holter monitoring before pacemaker implantation consistently influenced the modeling process for predicting clinically relevant AHREs using the three ML-based models (Table 3 and Fig. 4).

**Table 3:** Feature importance in the three machine learning-based models.

Rank	Random Forest		Support Vector Machine		eXtreme Gradient Boosting	
	Features	Importance*	Features	Importance*	Features	Importance*
1	Prior stroke/TIA	0.184	Beta adrenergic receptor blockers	0.254	Beta adrenergic receptor blockers	0.306
2	Beta adrenergic receptor blockers	0.178	Prior stroke/TIA	0.253	Prior stroke/TIA	0.158
3	> 1% APC on Holter, pre-implantation	0.145	> 1% APC on Holter, pre-implantation	0.220	LA diameter	0.132
4	Dyslipidemia	0.111	LA diameter	0.088	Age	0.105
5	LA diameter	0.104	Baseline systolic blood pressure	0.063	Baseline systolic blood pressure	0.075
6	QRS duration	0.101	Dyslipidemia	0.044	QRS duration	0.073
7	Baseline systolic blood pressure	0.085	Pacemaker indication	0.040	> 1% APC on Holter, pre-implantation	0.071
8	Age	0.057	QRS duration	0.030	Dyslipidemia	0.030
9	Pacemaker indication	0.030	Diabetes	0.005	Diabetes	0.028
10	Diabetes	0.006	Age	0.003	Pacemaker indication	0.023

*AAD* average absolute deviation, *APC* atrial premature contraction, *CI* confidence interval, *TIA* transient ischemic attack, *LA* left atrium.

* Feature importance was measured from sensitivity analysis.

## Discussion

In this prospective, multicenter, observational registry study, we found that the three ML algorithms (RF, SVM, and XGB) were better at identifying individuals who will develop clinically relevant AHREs among those with pacemaker implants. The XGB algorithm showed better performance and Brier scores than did the other algorithms. We also defined and calculated the index of feature importance of the variables in all three ML-based models. We found consistent feature importances for beta-adrenergic receptor blocker use, prior stroke or TIA, > 1% atrial premature contraction on Holter monitoring before pacemaker implantation, and LA diameter on echocardiography.

The detection of clinically relevant AHREs with cardiac implantable electronic devices is becoming increasingly attractive for predicting the progression to AF or reducing the risk of embolic stroke. Although various predictors of AHREs^4,8,27,28^ have been identified, they did not show highly consistent results. ML is powerful and has become ubiquitous and indispensable for solving complex medical problems. It offers an improved description and development of decision support tools to predict clinical events and encourage steps forward. Although it has been reported that AHREs ≥ 30 s or ≥ 2 min is associated with cerebrovascular events in previous studies^29,30^, other studies have reported the more relevant diagnostic points for AHREs (> 6 min)^1,31,32^. The current ESC guidelines also suggest that a minimum of 5 to 6 min of AHREs is associated with progression to AF, ischemic stroke, and major adverse cardiovascular events^3,5,6^.

To our knowledge, this is the first study to apply an ML algorithm to predict clinically relevant AHREs in patients with pacemaker implants and demonstrate improved prediction compared with that obtained with traditional statistical methods.

We selected the RF, SVM, and XGB algorithms and applied them to each predictive model in comparison to the logistic regression model. Because our dataset was relatively small and imbalanced, we used the five repeated tenfold CV method, instead of specifying a validation set in a training set separately, and the SMOTE to oversample the data set. In this study, we assessed the AUROC, AUPRC, F1-score, accuracy, sensitivity, specificity, NPV, and PPV and calculated the Brier score to evaluate the performance metrics of each model. The model with the XGB algorithm achieved the best performance. The predictive models also provide an opportunity to understand the features that may contribute to clinically relevant AHREs. This approach can identify the quantitation of feature importance for each variable, and we found consistent (beta-adrenergic receptor blocker use, prior stroke or TIA, > 1% atrial premature contraction on Holter monitoring before pacemaker implantation, and LA diameter) factors in the predictive models. Some of these features (prior stroke or TIA and LA diameter) have been described previously^8,16^.

This study had several strengths. The predictive models were based on simple and readily available clinical characteristics. Although the models appear only as mathematical exercise, it can provide information that is indirectly helpful to clinicians. Recently, Perino et al.^33^ reported that with increasing AHREs lasting for > 6 min to > 24 h, the stroke risk increased in those who did not receive anticoagulation treatment and mostly decreased in those who did. Vergara et al.^34^ investigated the temporal association between AHREs and the risk of ventricular arrhythmias (VA). AHREs that precede VA increased the risk of VA recurrence. In these regards, the predicted probability for AHREs lasting for > 6 min could be shared with the patient, and anticoagulation or antiarrhythmic therapy could serve as a critical element to prevent ischemic stroke or the recurrence of VA. The temporal association was observed We compared the model evaluation performance metrics, and the XGB model yielded greater discrimination and accuracy than did the other models and logistic regression. Meanwhile, the SVM model exhibited relatively worse performance metrics than did the RF and XGB models because the ensemble model tends to have better performance and robustness than the single model. Our study showed that ML algorithms may play a role in precision cardiology. Although the baseline characteristics of the derivation and validation sets were slightly different, the model performance metrics showed acceptable results.

This study has some limitations. As the data sets, especially the validation set, were relatively small and contained limited features, the current analysis findings may not necessarily be representative of the predictors of clinically relevant AHREs in patients with implanted pacemakers. However, the patients were prospectively enrolled from 11 tertiary centers, which can yield some degree of generalizability. In this study, 61.5% were women. Many women were enrolled, but the ratio of men and women was comparable to similar studies in the same region^35,36^. Further assessment and improvement of the applicability of the predictive models are necessary for larger and different-race populations.

Finally, the models developed herein used only clinically relevant AHREs as the outcome data, not progression to clinical AF, ischemic stroke, MACEs, and death. Further improvements in multiple outcome prediction with higher accuracy should be explored.

## Conclusion

Our study illustrated the utility of ML algorithms in estimating clinically relevant AHREs in patients with implanted pacemakers using easily obtainable preimplantation features. Classification of patients using these models can support clinical decisions for anticoagulation therapy to prevent adverse outcomes in selected patients. From this perspective, models need to be built and validated individually for each diagnosis. More high-quality evidence can be obtained by applying ML algorithms; consequently, the data obtained can aid in the optimal management of these patients with shared decision-making.

## Supplementary Information

Supplementary Information.

## Data Availability

The data that support the findings of this study are available from a web-based case report form on the Internet-based Clinical Research and Trial Management System (iCReaT), a data management system established by the Centers for Disease Control and Prevention, Ministry of Health and Welfare, Republic of Korea (iCReaT study no. C170004) but restrictions apply to the availability of these data, which were used under license for the current study, and so are not publicly available. Data are however available from the authors upon reasonable request and with permission of all investigators of the AF-Pacemaker study.
