# Supplementary material for: Artificial intelligence predicts clinically relevant atrial high-rate episodes in patients with cardiac implantable electronic devices
Source: Sci Rep. 2022 Jan 7;12:37. doi: 10.1038/s41598-021-03914-4 (PMC8741914; doi:10.1038/s41598-021-03914-4)

Supplementary Table 1. Correlation indices measured for correlation–based selection.

Discrete variables*		Continuous variables†	
**Variables**	**Correlation**	**Variables**	**Correlation**
Pacemaker indication	0.147	QRS duration	0.075
Beta adrenergic receptor blockers	0.124	Age	0.044
>1% APC on Holter, pre-implantation	0.104	LA diameter	0.041
Dyslipidemia	0.056	Baseline systolic blood pressure	0.040
Prior stroke/TIA	0.050	QTc interval	0.039
ARB/ACEi	0.044	Body mass index	0.021
Diabetes	0.042	LVEF	0.010
>1% VPC on Holter, pre-implantation	0.038	Baseline diastolic blood pressure	0.008
Statin	0.037	Baseline eGFR	0.002
Vascular disease	0.030	Baseline heart rate	0.002
Calcium channel blocker	0.015		
Sex	0.011		
Hypertension	0.011		
Smoking	0.006		
Heart failure	0.005		
Chronic kidney disease	<0.001		
Alcohol	<0.001		
Diuretics	<0.001		

Abbreviations: ACEi, angiotensin-converting-enzyme inhibitor; APC, atrial premature complex; ARB, angiotensin receptor blocker; GFR, glomerular filtration rate; LA, left atrium; LVEF, left ventricular ejection fraction; QTc, corrected QT; TIA, transient ischemic attack; VPC, ventricular premature complex
* Cramér's V was applied for discrete variables.
† Point-biserial correlation coefficient was applied for continuous variables

Supplementary Table 2. Description of the selected variables for the predictive model

Type of variables	Description
Continuous variables	QRS duration,<br>Age,<br>LA diameter,<br>Baseline systolic blood pressure
Discrete variables	Pacemaker indication,<br>Beta adrenergic receptor blockers,<br>>1% APC on holter, pre-implantation,<br>Dyslipidemia,<br>Prior stroke/TIA,<br>Diabetes

Abbreviation: APC, atrial premature contraction; LA, left atrium; TIA, transient ischemic attack

Supplementary Table 3. Baseline characteristics of the derivation and validation sets.

Variables	Derivation set (n=567)	Validation set (n=154)	p-value
**Demographic**			
Age, (years)	71 (66, 79)	73 (65, 79)	0.269
Male, n (%)	228 (40.2)	57 (37.0)	0.531
Body mass index, (kg/m^2)	24.2 (22.0, 26.6)	24.5 (22.2, 26.0)	0.589
Smoking, n (%)			
Former/Current	84 (14.8)	6 (3.9)	<0.001
Alcohol, n (%)	89 (15.7)	4 (2.6)	<0.001
Former/Current			
**Clinical**			
Heart failure, n (%)	19 (3.4)	7 (4.6)	0.645
Hypertension, n (%)	384 (67.7)	100 (64.9)	0.578
Diabetes, n (%)	154 (27.2)	42 (27.3)	1.000
Prior stroke/TIA, n (%)	65 (11.5)	15 (9.7)	0.646
Vascular disease, n (%)	50 (8.8)	21 (13.6)	0.104
Dyslipidemia, n (%)	205 (36.2)	17 (11.0)	<0.001
Chronic kidney disease, n (%)	50 (8.8)	9 (5.8)	0.304
**CHA_2DS_2VAS score***	3 (2, 4)	3 (2, 4)	0.259
**CHA_2DS_2VAS score, group**			0.396
0, n (%)	16 (2.8)	5 (3.2)	
1, n (%)	87 (15.3)	17 (11.0)	
≥ 2, n (%)	464 (81.8)	132 (85.7)	
Pacemaker indication			0.071
Sick sinus syndrome, n (%)	235 (41.4)	77 (50.0)	
AV block, n (%)	332 (58.6)	77 (50.0)	
Baseline systolic blood pressure, (mmHg)	135 (122, 142)	135 (121, 149)	0.199
Baseline diastolic blood pressure, (mmHg)	70 (63, 79)	72 (64, 81)	0.020
Baseline heart rate, (/min)	60 (46, 70)	60 (53, 73)	0.123
Baseline eGFR, (mL/min/1.73 m^2)	73.0 (60.3, 86.0)	78.0 (63.0, 94.0)	0.011
**Electrocardiogram**			
QRS duration, (ms)	98 (84, 128)	110 (90, 146)	<0.001
QTc interval, (ms)	439 (411, 467)	460 (427, 491)	<0.001
**Echocardiography**			
LA diameter, (mm)	42 (36, 46)	40 (35, 45)	0.009
LVEF, (%)	63 (60, 68)	66 (61, 70)	0.001
**Holter recording**			
APC >1% at pre-implantation, n (%)	73 (7.6)	10 (6.5)	0.775
VPC >1% at pre-implantation, n (%)	39 (6.9)	10 (6.5)	1.000
**Medications**			
ARB/ACEi, n (%)	241 (42.5)	71 (46.1)	0.479
Beta adrenergic receptor blocker, n (%)	87 (15.3)	25 (16.2)	0.885
Calcium channel blocker, n (%)	174 (30.7)	50 (32.5)	0.745
Statin, n (%)	245 (43.2)	61 (39.6)	0.478
Diuretics, n (%)	131 (23.1)	31 (20.1)	0.499

The data are presented as number (%), median [IQR]

Abbreviations: ACEi, angiotensin-converting-enzyme inhibitor; AHREs, atrial high-rate episodes; APC, atrial premature complex; ARB, angiotensin receptor blocker; AV, atrioventricular; GFR, glomerular filtration rate; LA, left atrium; LVEF, left ventricular ejection fraction; QTc, corrected QT; TIA, transient ischemic attack; VPC, ventricular premature complex

*The CHA_2DS_2-VAS score is a measure of the risk of stroke in patients with atrial fibrillation, with scoring ranging from 0 to 9 and higher scores indicating greater risk. Congestive heart failure, hypertension, age 75 years or older (doubled), diabetes, stroke (doubled), vascular disease, age 65 to 74 years, sex category (female)

Supplementary Figure 1. Discrimination between informative versus non-informative variables

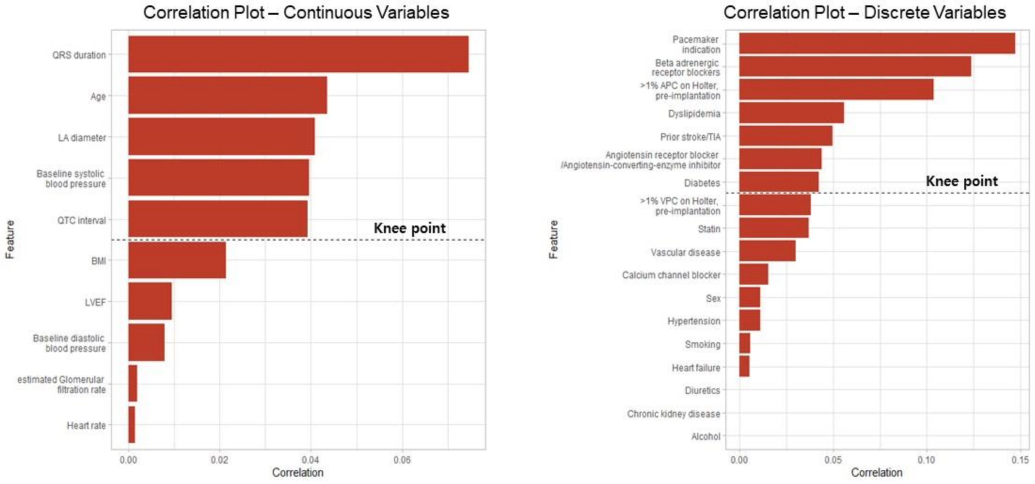

Supplementary Figure 2. RF model for predicting clinically relevant AHREs in patients with pacemaker

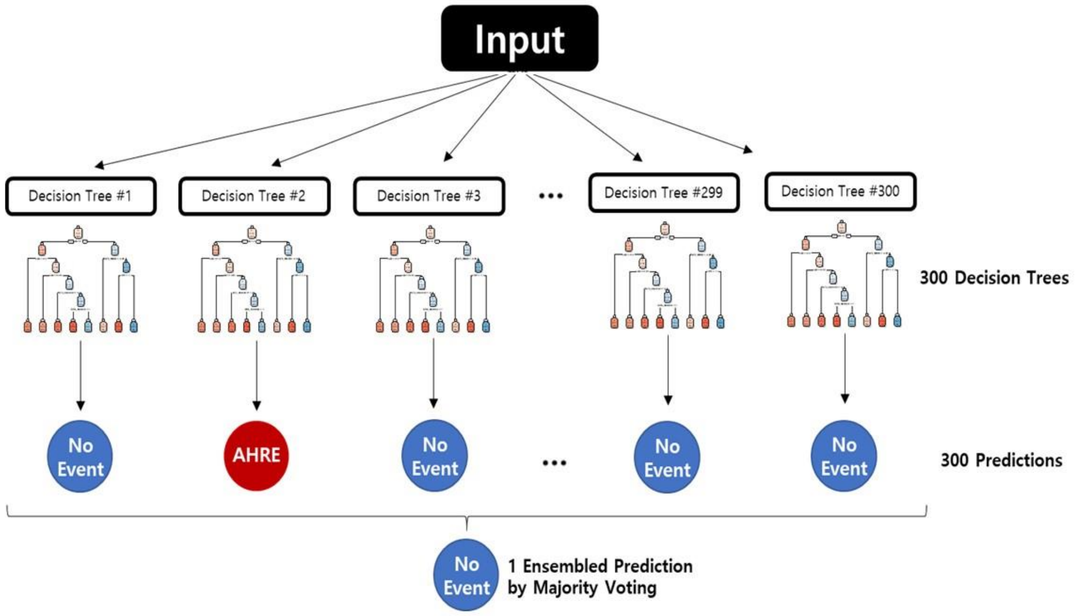

Supplement: Supplementary file 1 — Supplementary Information. [file 41598_2021_3914_MOESM1_ESM.pdf]
